# Metabolite Production and Extraction of Indole Compound From the Tomato Endophyte *Streptomyces* sp. VITGV100

**DOI:** 10.21769/BioProtoc.5386

**Published:** 2025-07-20

**Authors:** Veilumuthu Pattapulavar, Sathiyabama Ramanujam, Sanjivkumar Muthusamy, Shweta Panchal, John Godwin Christopher

**Affiliations:** 1 Department of Biomedical Sciences, School of BioSciences and Technology, Vellore Institute of Technology, Vellore, India; 2 Department of Science and Humanities, Karpagam Academy of Higher Education, Coimbatore, Tamil Nadu, India; 3 Department of Microbiology, K.R. College of Arts & Science, K.R. Nagar, Kovilpatti, Tamil Nadu, India; 4 Department of Integrative Biology, School of BioSciences and Technology, Vellore Institute of Technology, Vellore, Tamil Nadu, India

**Keywords:** Endophytic *Streptomyces*, Whole-genome sequencing, Secondary metabolites, Antibacterial activity, Biosynthetic gene clusters, Comparative genomics, GC–MS analysis, *Lycopersicon esculentum*

## Abstract

Endophytic actinomycetes, particularly *Streptomyces* species, have gained significant attention due to their potential to produce novel bioactive compounds. In this study, we isolated and characterized an endophytic *Streptomyces* sp. VITGV100 from the tomato plant (*Lycopersicon esculentum*), employing the direct streak method and whole-genome sequencing. A genome analysis was done to uncover its biosynthetic potential and identify indole-type compounds. The strain's secondary metabolite production was evaluated through GC–MS analysis, and its antimicrobial activity was tested against selected human pathogenic bacteria. Our protocol outlines a comprehensive approach, describing the isolation and extraction of metabolites and genome mining for indole-type compounds. This isolate has potential pharmaceutical applications, accelerating the discovery of novel indole-type bioactive compounds.

Key features

• A systematic approach for isolating *Streptomyces* sp. VITGV100 from *Lycopersicon esculentum*, including surface sterilization and selective culturing on ISP2 medium.

• Whole-genome sequencing and annotation: High-throughput Illumina sequencing followed by genome assembly, annotation, and functional characterization to explore biosynthetic potential and metabolic pathways.

• Secondary metabolite profiling and bioactivity screening: GC–MS-based identification of bioactive metabolites, followed by antibacterial activity assessment against human pathogens using well-diffusion assays.

• Identification of biosynthetic gene clusters (BGCs) using antiSMASH 6.0.

## Graphical overview


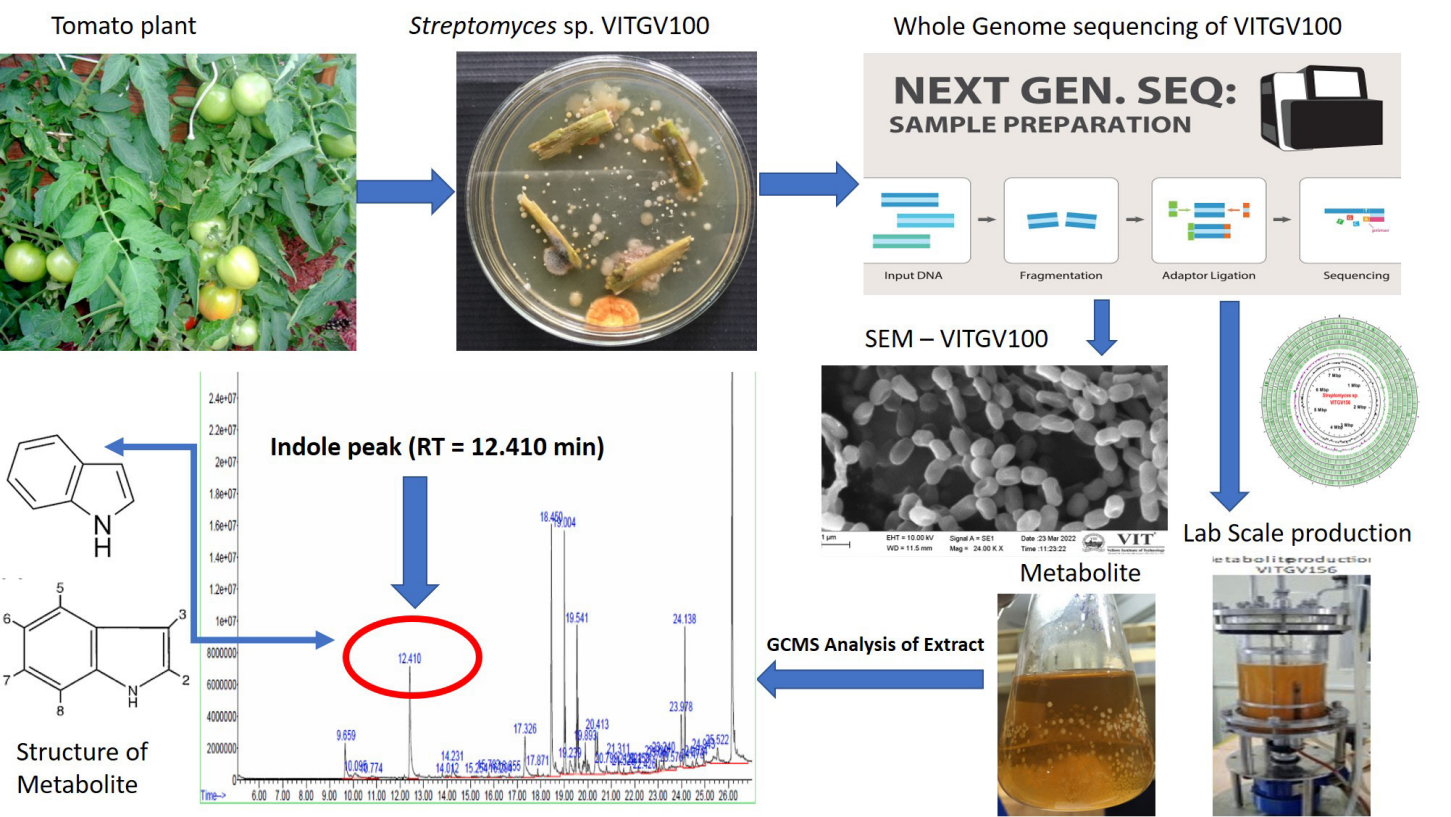


**Genomic and metabolomic insights into the isolation of endophytic *Streptomyces sp.* VITGV100: a potential source of bioactive indole compounds**

## Background

Actinomycetes, particularly those belonging to the genus *Streptomyces*, are a prolific source of bioactive secondary metabolites with significant applications in medicine, agriculture, and industry. Among them, *Streptomyces* species have garnered attention for their potential to produce novel antimicrobial, anticancer, and even plant growth-promoting compounds [1]. Microbial strains offer a sustainable and environmentally friendly alternative to synthetic agrochemicals, playing a crucial role in biocontrol and plant health enhancement [2]. Traditional methods for isolating *Streptomyces* from environmental sources involve selective culturing techniques using specific growth media, followed by morphological and biochemical characterization [3]. However, recent advances in molecular biology and whole genome sequencing (WGS) have revolutionized the identification and functional analysis of these microorganisms [4]. WGS enables comprehensive insights into the genetic architecture of *Streptomyces*, facilitating the discovery of biosynthetic gene clusters (BGCs) responsible for secondary metabolite production [5]. This genomic approach overcomes the limitations of conventional screening methods, which often fail to detect cryptic or silent gene products under standard laboratory conditions [6]. The protocol described herein focuses on the isolation, genome sequencing, and functional characterization of *Streptomyces* sp. VITGV100, an endophytic actinomycete strain isolated from *Lycopersicon esculentum* (tomato) plants [5]. Compared to traditional bioactivity-guided screening, this genomic approach enhances the efficiency of novel bioactive compound discovery by directly linking genetic information to metabolite production [7]. A key advantage of this protocol is its ability to systematically identify and analyze BGCs involved in secondary metabolite biosynthesis using tools such as antiSMASH [8] and ARTS [9]. This computational approach significantly accelerates the identification of novel antibiotics [10]. Furthermore, comparative genomic analyses using ANI and dDDH facilitate the taxonomic positioning and evolutionary relationships of the newly isolated strain, contributing to a deeper understanding of *Streptomyces* diversity and genetic variability [11]. Beyond antibiotic discovery, the protocol has broad applications in biotechnology and agriculture. Endophytic *Streptomyces* strains have been reported to enhance plant growth through phytohormone production, nitrogen fixation, and antagonism against phytopathogens [12]. In summary, this protocol represents a comprehensive strategy for isolating, characterizing, and harnessing the biosynthetic potential of endophytic *Streptomyces* strains.

## Materials and reagents

**Biological materials**

1. Healthy 4-week-old tomato plants grown in natural soil under conditions (*Lycopersicon esculentum*) (Arka Rakshak)

2. *Streptomyces* sp. VITGV100 (MCC 4961)

4. *Staphylococcus aureus* (MTCC 737)

5. *Escherichia coli* (MTCC 1687)

**Reagents**

1. International *Streptomyces* Project -2 agar (Himedia, CAS number: 8002-48-0)

2. Tetracycline (Himedia, CAS number: 64-75-5)

3. Cetyltrimethylammonium bromide (Himedia, AS number: 57-09-0)

4. Cyclohexamide (Himedia, CAS number: 66-81-9)

5. Nystatin (Himedia, CAS number: 1400-61-9)

6. Streptomyces DNA Purification kit (Himedia, CAS number: MBA527)

7. Ethanol (Himedia, CAS number: 64-17-5)

8. NaHCO_3_ (Himedia, CAS number: 144-55-8)

9. Ethyl acetate (Himedia, CAS number: 141-78-6)

10. Methanol (Himedia, CAS number: 67-56-1)

11. Glycerol (Himedia, CAS number: 56-81-5)

12. Sodium hypochlorite (Himedia, CAS number: 7681-52-9)

13. Mueller Hinton agar (MHA) (Himedia, CAS number: M1084)

**Solutions**

1. Yeast malt agar - ISP2 agar (see Recipes)

2. MHA agar (see Recipes)

**Recipes**

**1. Yeast malt agar - ISP2 agar**

**Table:** 

Ingredients	Final concentration
Peptone	5 g/L
Yeast extract	3 g/L
Malt extract	3 g/L
Dextrose (glucose)	10 g/L
Agar	20 g/L

**2. MHA agar**

**Table:** 

Ingredients	Final concentration
HM infusion solids B # (from 300 g)	2 g/L
Acicase	17.5 g/L
Starch	1.5 g/L
Agar	17. g/L

**Laboratory supplies**

1. Erlenmeyer flasks (Borosil Scientific, catalog number: 4980)

2. Separating funnel (Borosil Scientific, catalog number: 6403)

3. Round-bottom flask (Borosil Scientific, catalog number: 4380)

4. Storage bottle (Borosil Scientific, catalog number: 1519)

5. Sample vials (Borosil Scientific, catalog number: 9913)

6. Petri plates (Borosil Scientific, catalog number: 3165)

7. Cork borer (HiMedia, India: LA737)

## Equipment

1. Incubator (Thermo Scientific, catalog number: 50125590)

2. Hot air oven (Thermo Scientific, catalog number: PR305225M)

3. Biological safety cabinet, HEPA filter (Thermo Scientific, catalog number: 51025411)

4. Rotary vacuum evaporator (Fisher Scientific, catalog number: 05-405-195)

5. Gas chromatography and mass spectrophotometry (Thermo Scientific, model: TOEXGFTH)

6. Nanodrop (Thermo Scientific, model: ND-2000C)

7. Centrifuge (Remi, model: R-24)

8. Cooling centrifuge (Remi, model: CM-12)

9. Zone measuring scale (HiMedia, India, model: PW297)

10. GC–MS (Thermo Scientific Trace GC Ultra & ISQ Single Quadrupole MS, USA)

## Software and datasets

1. antiSMASH 6.0 (Manufacturer, Version - https://antismash.sondarymetabolites.org)

2. Alignment Search Tool (BlastX): https://blast.ncbi.nlm.nih.gov/Blast.cgi

3. Kyoto Encyclopedia of Genes and Genomes (KEGG):

3. KEGG Automatic Annotation Server: https://www.genome.jp/tools/kaas/

4. CGView server 1.0: https://proksee.ca/

5. Blast2GO platform: https://www.blast2go.com

6. Web Gene Ontology Annotation plot (http://wego.genomics.org.cn/cgi-bin/wego/index.pl)

7. MegaX 6.0: https://www.megasoftware.net/

8. Antimicrobial Resistant Target Seeker: https://arts.ziemertlab.com

9. Prokka (version 1.12): https://github.com/tseemann/prokka

10. SPAdes assembler (v-3.13.0): https://github.com/ablab/spades

11. Trimmomatic v0.38: http://www.usadellab.org/cms/?page=trimmomatic

12. *Streptomyces* sp. VITGV100: https://trace.ncbi.nlm.nih.gov/Traces/?view=run_browser&acc=SRR15294182&display=analysis

## Procedure

**A. Isolation of endophytic Streptomyces sp.**

1. Collect healthy tomato plants (*Lycopersicon esculentum*). In our work, healthy 4-week-old tomato plants *(L. esculentum)*, from Madurai (9.9420° N, 77.9724° E), India, grown in natural soil in an agricultural field (37 ± 2 °C, 12 h light/dark photoperiod), were used for endophyte isolation (lower stem 3–5 cm, plant measured upward from the soil surface).

2. Perform surface sterilization of plant samples using stems collected from a 4-week-old tomato plant. Cut the stems into 4–5 cm long segments using sterile surgical blades and do the following:

a. Immerse in 70% ethanol for 1 min.

b. Immerse in 90% ethanol for 1 min.

c. Treat with 0.9% sodium hypochlorite for 4 min.

d. Rinse in 70% ethanol for 30 s.

e. Soak in 10% NaHCO_3_ for 5 min.

f. Rinse thoroughly three times with sterile distilled water.

**Figure 1.**
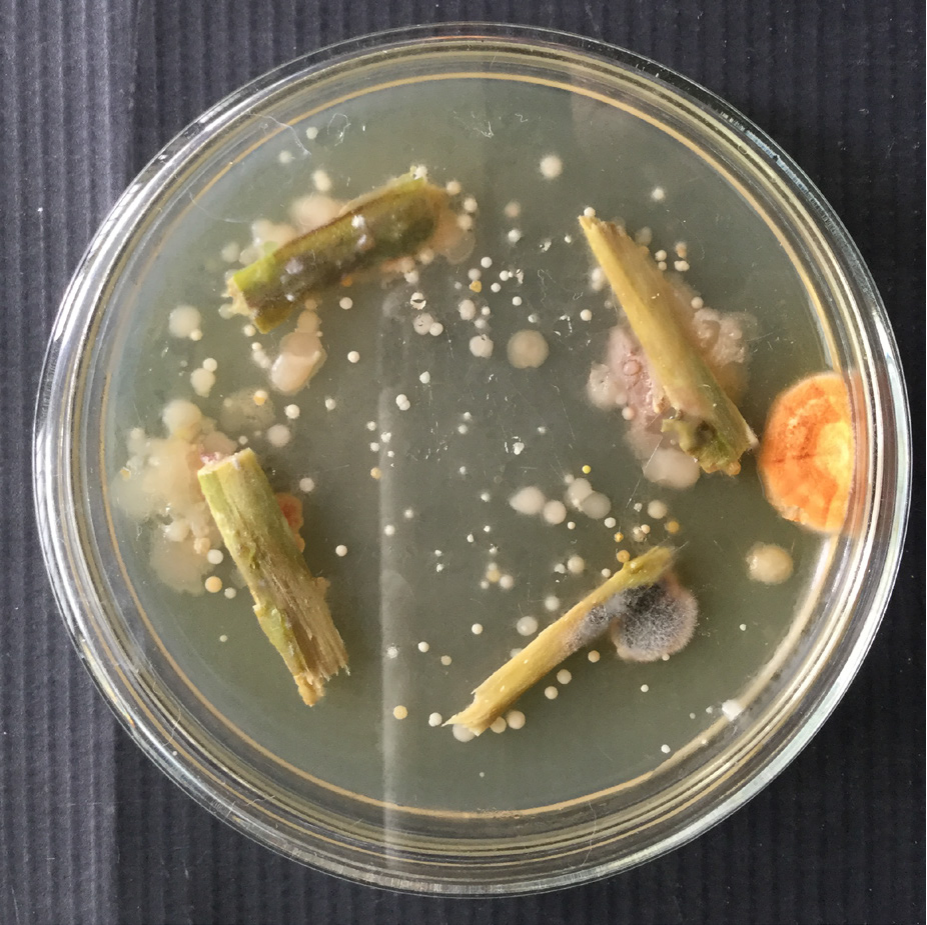
Isolation of endophytic *Streptomyces* sp. VITGV100

3. Cut the surface-sterilized lower stems in a cross direction and streak on ISP2 agar (pH 7.2 before autoclaving at 121 °C, 15 psi for 15 min) medium supplemented with cyclohexamide and nystatin (each used at 50 μg/mL in ISP2 agar). Stock solutions (10 mg/mL) were prepared in ethanol and filter-sterilized before use.

4. Then, incubate the plates at 30 °C for 15 days and isolate individual microbial colonies. All incubation steps should be carried out in a light/dark cycle at 30 °C to support endophyte growth. From this point onward, all procedures should be conducted under a clean bench/laminar flow hood to maintain sterility

5. After 15 days, pick distinct colonies with a powdery morphology and subculture onto fresh ISP2 agar plates to ensure purity and morphological consistency (Figure 1).

**B. Whole-genome sequencing**

**Figure 2.**
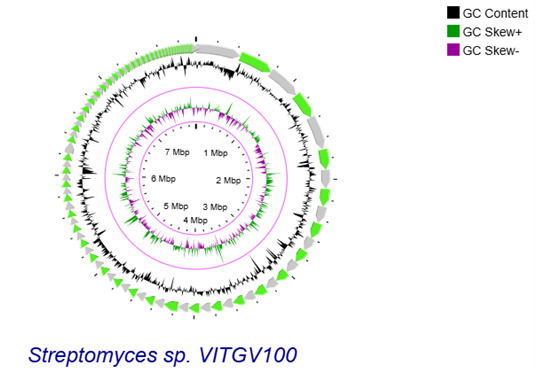
Genomic landscape of VITGV100. Circular genomic map of *Streptomyces* sp. VITGV100 assembled contigs are depicted as green arrows, accompanied by positive and negative GC skews indicated in the inner circle

1. Grow subcultures in ISP2 broth at 30 °C for 7 days with shaking at 120 rpm.

2. Extract genomic DNA using the CTAB method (Veilumuthu et al., 2022).

3. Assess DNA quality and quantity using NanoDrop. Typical DNA concentration is 146.9 ng/μL.

4. Prepare paired-end sequencing libraries using Illumina TruSeq Nano DNA Library Prep kit. In our case, paired-end sequencing was performed with 2 × 150 bp reads, achieving ~100× coverage. (Genome sequence deposited in NCBI; SRA accession number SRS9635924.)

5. Process sequencing data using Trimmomatic v0.38 to obtain high-quality reads. Perform quality trimming of raw reads using Trimmomatic v0.38 to remove adapter sequences and low-quality regions. Recommended command line (paired-end data): https://github.com/usadellab/Trimmomatic.

6. Assemble genome using SPAdes assembler v3.13.0. Assemble the cleaned reads using SPAdes v3.13.0 with default settings and careful flags to reduce mismatches: https://github.com/ablab/spades.

7. Perform annotation using Prokka v1.12 and identify rRNA genes. Annotate the assembled genome using Prokka v1.12 for gene prediction and identification of rRNA, tRNA, and CDS: https://github.com/tseemann/prokka.

8. Conduct functional annotation using the NCBI non-redundant protein database (BlastX) and KEGG database (KAAS). Do pathway mapping using KEGG Automatic Annotation Server: https://www.genome.jp/tools/kaas/.

9. Construct a circular genome using CGView server 1.0 (Figure 2).

10. All analyses were run in Ubuntu Linux, with Trimmomatic and SPAdes via command line, and Blast2GO on a Java-supported desktop client. The genome sequencing was done with Eurofins Genomics Pvt. Ltd, and results were compiled.

**C. Secondary metabolite gene cluster analysis**

**Figure 3.**
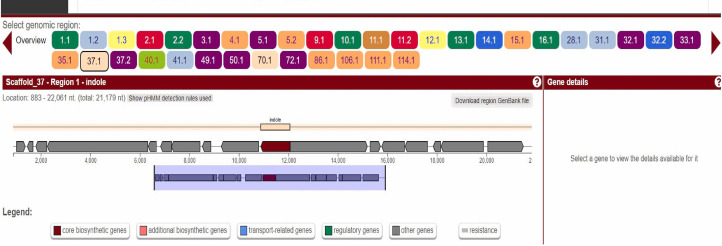
Screenshot highlighting two major stions: core gene table prioritization and visualization of the BGC of indole located in scaffold 37.1 region of the genome

**Figure 4.**
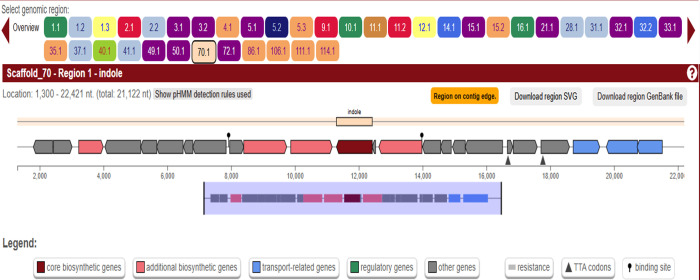
Screenshot highlighting two major stions: core gene table prioritization and visualization of BGC of indole located in scaffold 70.1 region of the genome

1. Detect biosynthetic gene clusters (BGCs) using antiSMASH 6.0 (Figures 3 and 4).

2. The biosynthetic potential of *Streptomyces* sp. VITGV100 was analyzed using antiSMASH 6.0, which identified multiple secondary metabolite biosynthetic gene clusters, as summarized in Table 1.

3. Utilize ARTS for antibiotic-specific genome mining.

**Table 1.** Secondary metabolite cluster of *Streptomyces* sp. VITGV100 obtained using antiSMASH 6.0

S.No	Region	Type	Most similar known cluster	Similarity
1	Region 1.1	NRPS	Coelichelin	100%
2	Region 1.2	NRPS, lanthipeptide-class-I	Coelibactin	90%
3	Region 1.3	lanthipeptide-class-III	Sapb	100%
4	Region 2.1	Siderophore	Paulomycin	9%
5	Region 2.2	NRPS	Cda1b/Cda2aCda3a	72%
6	Region 3.1	Terpene	Lysolipin I	4%
7	Region 4.1	T3PKS	Herboxidiene	8%
8	Region 5.1	Terpene	Hopene	100%
9	Region 5.2	T1PKS	Streptovaricin	31%
10	Region 9.1	Siderophore	–	–
11	Region 10.1	NRPS-like	Streptothricin	95%
12	Region 11.1	Melanin	Melanin	60%
13	Region 11.2	Siderophore	Desferrioxamin B/Desferrioxamine E	83%
14	Region 12.1	Lanthipeptide-ClassIII	Catenulipeptin	60%
15	Region 14.1	RiPP-like	–	–
16	Region 15.1	T2PKS	Spore Pigment	66%
17	Region 16.1	NRPS-like	Alanylclavam/2	12%
18	Region 28.1	PKS-like, class-V	Methylenomycin A	9%
19	Region 31.1	T1PKS, NRPS, NRPS like	Candicidin	90%
20	Region 32.1	Terpene	Versipelostatin	5%
21	Region 32.2	RiPP-like	Informatipeptin	42%
22	Region 33.1	Terpene	Geosmin	100%
23	Region 35.1	T1PKS	Nystatin A1	31%
24	Region 37.1	Indole	7-Prenylisatin	100%
25	Region 37.2	Terpene	Isorenieratene	100%
26	Region 40.1	Ectoine	Ectoine	100%
27	Region 1.1	PKS-like, T2PKS, Butyrolactone	Fluostatins M-Q	69%
28	Region 49.1	Terpene	Isorenieratene	18%
29	Region 50.1	Terpene	Carotenoid	45%
30	Region 70.1	Indole	5-Isoprenylindole-3-Carboxylate Β-D Glycosyl Ester	23%
31	Region 72.1	Terpene	Albaflavenone	100%
32	Region 86.1	hglE-KS	2'-Chloropentostatin	6%
33	Region 106.1	T1PKS	Griseochelin	53%
34	Region 111.1	T1PKS	Sanglifehrin A	11%
35	Region 114.1	T1PKS	–	–

NRPS: non-ribosomal peptide synthetase.

T1PKS: Type 1 Polyketide synthases.

T2PKS: Type 2 Polyketide synthases.

T3PKS: Type 3 Polyketide synthases.

RiPP: Ribosomally synthesized and post-translationally modified peptides.

**D. Gene ontology analysis**

1. Perform GO annotations using Blast2GO platform. Gene ontology enrichment visualization:

http://wego.genomics.org.cn/.

2. Categorize gene functions into biological process (BP), molecular function (MF), and cellular component (CC).

3. Retrieve GO terms using WEGO portal and Blast2GO.

**E. Metabolite extraction**

**Figure 5.**
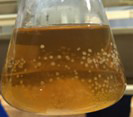
Visual inspection of pellets and aggregates of *Streptomyces* sp. VITGV100

**Figure 6.**
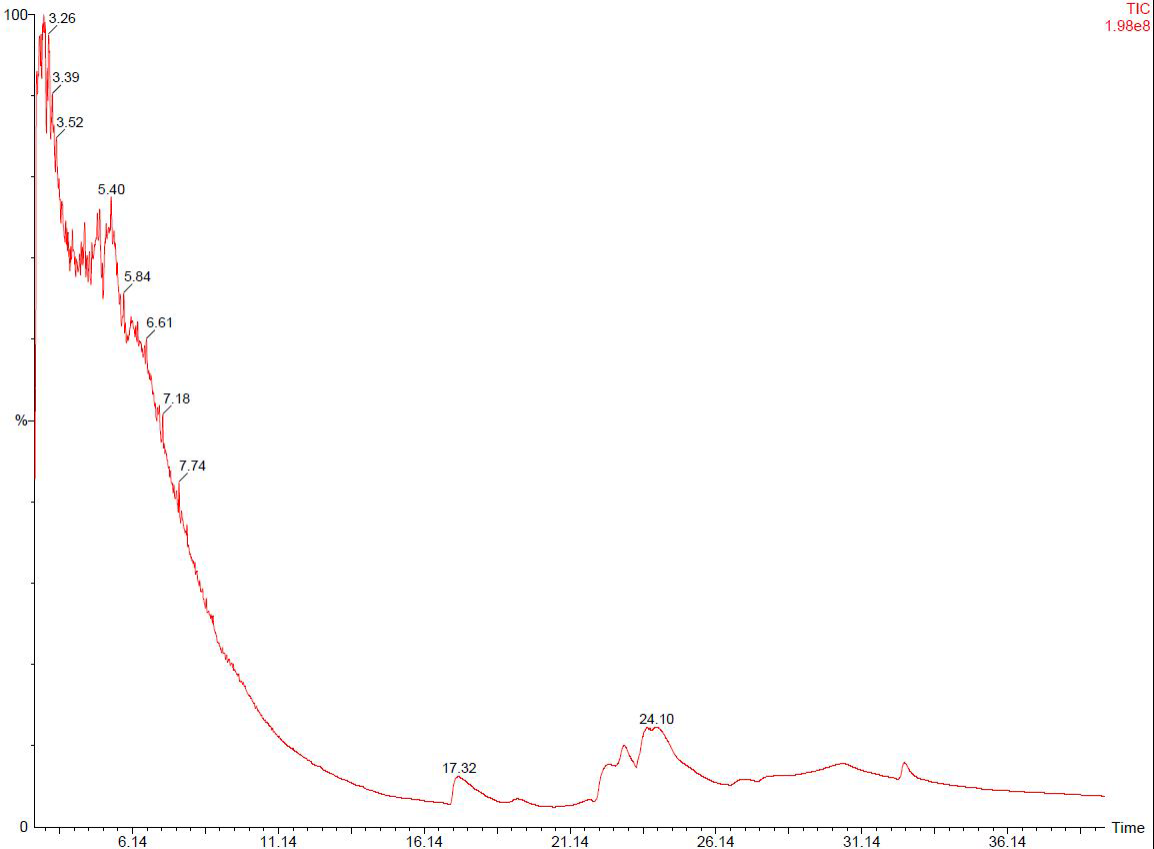
Blank media extract was run as an ISP2 control during GC–MS analysis

1. Grow subcultures in ISP2 broth at 30 °C for 15 days with a 1:50 inoculum ratio and shaking at 120 rpm.

2. Confirm cellular growth by visual inspection of pellets and aggregates (Figure 5).

3. Centrifuge the culture broth at 100 × g for 20 min at 4 °C and collect the supernatant in a separate tube. Discard cells.

4. To the supernatant, add an equal volume of ethyl acetate and shake the mixture vigorously for 10 min.

5. Incubate the flask in a rotary shaker at 200 rpm for 24 h at room temperature to enhance the extraction process. Bead beating is not required, but may enhance extraction efficiency.

6. Allow the mixture to settle undisturbed for 30 min, enabling phase separation.

7. Carefully collect the upper organic phase containing the metabolites and concentrate it using a rotary evaporator at 54 °C and 80 rpm until a dry or semi-solid residue is obtained. Collect organic extracts in sterile glass vials to avoid solvent interaction with plastic.. After drying the solvent in a rotary evaporator, weigh the resulting residue.

8. Dissolve the dried crude extract in 200 μL of acetonitrile to ensure proper solubility and store it at -20 °C for further use.

9. Blank media extract was included as an ISP2 control to validate compound specificity during GC–MS analysis, as shown in Figure 6.

**F. GC–MS analysis**

1. Analyze crude extract using GC–MS.

2. Use a TG-5MS fused silica capillary column (30 m × 0.25 mm × 0.1 mm film thickness).

3. Set electron ionization at 70 eV and helium flow at 1 mL/min.

4. Maintain the injector and MS transfer line temperature at 280 °C.

5. Use the following temperature program:

a. 50 °C (2 min hold).

b. Increase to 150 °C at 7 °C/min.

c. Increase to 270 °C at 5 °C /min.

d. Increase to 310 °C at 3.5 °C/min.

**Figure 7.**
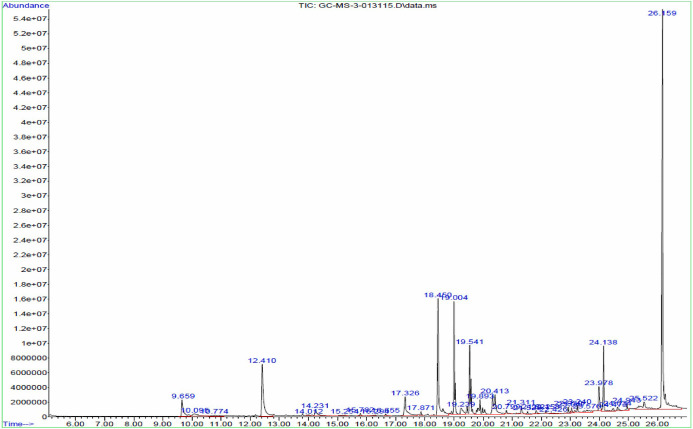
GC–MS chromatogram obtained from the crude extract of *Streptomyces* sp. VITGV100

**Figure 8.**
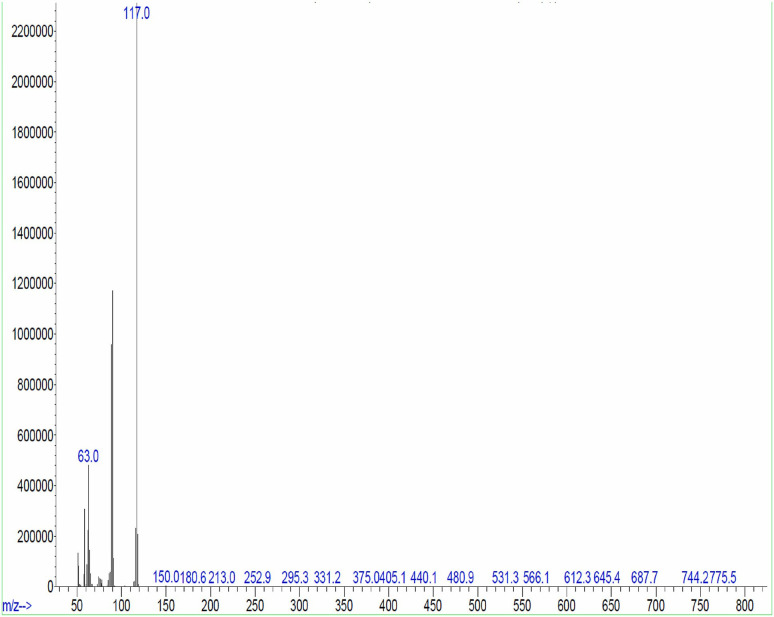
Indole massspectrum obtained from the crude extract of *Streptomyces* sp. VITGV100 at 12.40 min

The major chemical constituents present in the crude extract of *Streptomyces* sp. VITGV100, as identified by GC–MS analysis, are listed in Table 2. GC–MS analysis revealed that the ethyl acetate extract of *Streptomyces* sp. VITGV100 contained 35 metabolites. Indole was detected at 12.410 min. The identity of each compound was confirmed using the NIST mass spectrometry database. Figure 7 presents the GC–MS chromatogram obtained from the crude extract. Figure 8 shows the mass spectrum of indole obtained from the crude extract.

**Table 2.** Major chemical compounds identified by GC–MS for *Streptomyces* sp. VITGV100

S. No	Chemical compound	RT	Molecular weight	Molecular formula	Area %	Activity	Ref
1	Ethanol, 2-(2-butoxyethoxy)	9.659	162.23	C_8_H_18_O_3_	2.54	-	
2	Butyl 2-(2-(2-ethoxyethoxy) ethoxy) acetate	10.095	**248.3160**	C_12_H_24_O_5_	1.02	-	
3	3-Aminobenzhydrazide	10.774	**151.1659**	C_7_H_9_N_3_O	0.4	Antimicrobial	[1]
4	Indole	12.410	117.148	C_8_H_7_N	7.05	Antimicrobial	[2]
5	Bicyclo[4.2.0]octa-1,3,5-triene-7-carboxylic acid	17.024	**104.1491**	C_8_H_8_	0.16	-	-
6	Benzeneethanol, 4-hydroxy	14.231	**138.1638**	C_8_H_10_O_2_	1.33	Antimicrobial	[3]
7	1-Penten-3-yne, 2-methyl	15.254	**80.1277**	C_6_H_8_	0.31	-	-
8	5-Eicosene, (E)	15.782	280.5316	C_20_H_40_	0.24	-	-
9	3-Methylbut-2-enoic acid, 8-chlorooctyl ester	16.294	**128.171**	C_13_H_23_ClO_2_	0.18	-	-
10	Acetophenone, 4'-amino-	16.655	135.1632	C_8_H_9_NO	0.21	Antimicrobial	[4]
11	Benzoic acid, 4-(methylamino)-	17.326	151.16	C_8_H_9_NO_2_	3.26	Antimicrobial	[5]
12	5-Eicosene, (E)-	17.871	280.5316	C_20_H_40_	0.24	Antimicrobial	[6]
13	n-Hexadecanoic acid	18.450	**256.4241**	C_16_H_32_O_2_	10.34	Antimicrobial	[7]
14	1,2-Benzenedicarboxylic acid, bis(2-methylpropyl) ester	19.004	**278.3435**	C_16_H_22_O_4_	7.16	Antimicrobial	[8]
15	(p-Hydroxyphenyl) glyoxal	19.239	150.13	C_8_H_6_O_3_	1.32	Antimicrobial	[9]
16	Dibutyl phthalate	19.541	278.3435	C_16_H_22_O_4_	6.07	Antimicrobial	[10]
17	Phthalic acid, isobutyl octyl ester	19.893	**334.456**	C_20_H_30_O4	2.80	Antimicrobial	[11]
18	1,2-Benzenedicarboxylic acid, butyl octyl ester	20.413	**334.456**	C_20_H_30_O_4_	4.65	Antimicrobial	[12]
19	1,2-Benzenedicarboxylic acid, bis(1-methylethyl) ester	20.799	**250.2903**	C_14_H_18_O_4_	0.44	-	-
20	Hexadecanamide	21.311	311.5	C_16_H_33_NO	0.89	-	-
21	Cyclotetracosane	21.529	336.6380	C_24_H_48_	0.20	-	-
22	cis-11-Hexadecenal	21.822	238.41	C_16_H_30_O	0.46	-	-
23	4-s-Butylaniline	22.158	149.233	C_10_H_15_N	0.24	-	-
24	1,6-Dibromo-2-cyclohexylpentane	22.426	**444.12**	C_11_H_20_Br_2_	0.16	-	-
25	1H-Indole, 3-methyl-	22.930	**174.2423**	C_11_H_14_N_2_	0.63	Antimicrobial	
26	Octadecanamide	23.047	536.0	C_18_H_37_NO	0.39	-	-
27	Phenol, 2-propyl-	23.240	**136.1910**	C_9_H_12_O	0.73	-	-
28	Octadecanoic acid, octadecyl ester	23.576	**536.9557**	C_36_H_72_O_2_	0.91	-	-
29	Octadecanoic acid, 2,3-dihydroxypropyl ester	23.978	**358.5558**	C_21_H_42_O _4_	2.14	-	-
30	Diisooctyl phthalate	24.138	**390.56**	C_24_H_38_O_4_	4.09	-	-
31	8-Quinolinamine	24.473	144.17	C_9_H_8_N_2_	0.30	Antimicrobial	
32	cis-11-Eicosenamide	24.624	**309.53**	C_20_H_39_NO	0.45	-	-
33	Dodecanoic acid, 2-butoxyethyl ester	24.943	**285.49**	C_18_H_36_O_3_	0.57	-	-
34	Methyl 11-oxo-9-undecenoate	25.522	212.28	C_12_H_20_O_3_	2.60	-	-
35	9-Octadecenamide, (Z)-	26.159	3281.476	C_18_H_35_NO	35.47	-	-

**G. Secondary screening against human pathogens**

**Figure 9.**
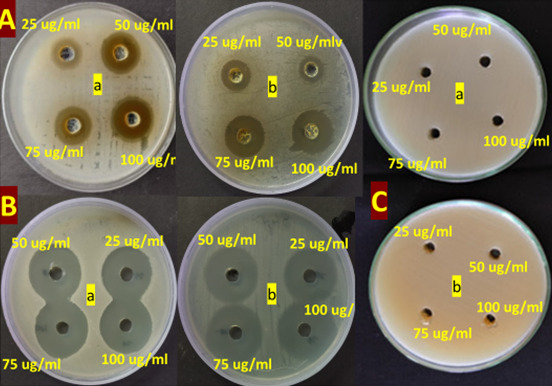
Secondary screening of culture extract from *Streptomyces* sp. VITGV100 against (a) *S. aureus* and (b) *E. coli* at different concentrations (25, 50, 75, and 100 µg) by the well-diffusion method

1. Cut 6 mm diameter wells in agar plates using a sterile steel cork borer.

2. Compare the antibacterial activity of the crude extract with tetracycline (positive control).

3. Prepare four different concentrations (25, 50, 75, and 100 μg/mL) of the crude extract in ethyl acetate and add to 4 different wells (100 μL each). Include negative controls (ethyl acetate) and positive controls (tetracycline) in all experiments.

4. Spread 100 µL of bacterial culture (OD600 ~0.6) evenly using a sterile cotton swab to create a confluent lawn. Air-dry plates for 10 min before well diffusion.

5. Prepare each test plate with a uniform lawn of the respective test bacteria, including *Escherichia coli* and *Staphylococcus aureus*, to evaluate the antibacterial activity of the crude extract.

6. Incubate plates at 37 °C for 24 h.

7. The antimicrobial activity of the crude extract was evaluated against selected test organisms (Figure 9), and the results, expressed as mean inhibition zone diameters (± SD, n = 3), are presented in Table 3.

**Table 3.** Antimicrobial activity of crude extract and controls (mean ± SD, n = 3)

Strain	Treatment	25 µg/mL	50 µg/mL	75 µg/mL	100 µg/mL
***E. coli***	VITGV38 extract	15 ± 0.47	16 ± 0.94	17 ± 0.47	18 ± 0.82
	Tetracycline	23 ± 1.41	24 ± 0.82	24 ± 0.47	25 ± 1.25
	Ethyl acetate	–	–	–	–
***S. aureus***	VITGV38 extract	10 ± 0.94	14 ± 0.47	15 ± 0.82	17 ± 0.94
	Tetracycline	16 ± 0.47	18 ± 1.25	19 ± 0.94	19 ± 1.25
	Ethyl acetate	–	–	–	–

## Data analysis

All experimental data were processed using standardized statistical and bioinformatics approaches. Data points deviating by more than 2 standard deviations from the mean were considered outliers and excluded. Only data from experiments with at least three independent biological replicates were included. Technical replicates with >10% variation was removed from further analysis. Biological replicates: Minimum of three independent replicates per experimental condition. Technical replicates: Minimum of three technical replicates per sample for reproducibility.

Data analysis was performed using the following software tools:

MEGA X: Used for phylogenetic analysis.

Linux (Ubuntu 20.04): Required for running bioinformatics pipelines such as SPAdes genome assembly, Prokka annotation, and antiSMASH BGC detection.

## Validation of protocol

This protocol has been rigorously tested to ensure reproducibility and robustness across multiple independent experiments.

1. Experimental validation

All experiments were performed with a minimum of three biological replicates and three technical replicates per sample. Negative controls (untreated samples) and positive controls (reference standards) were included in all experiments.

2. Reproducibility and consistency

Independent validation experiments were conducted under identical conditions, yielding consistent colony morphology, metabolite production, and genome sequencing results. Whole-genome sequencing quality metrics (e.g., N50 values, read coverage, and BUSCO completeness scores) confirmed the reliability of the sequencing and assembly steps. Metabolite profiling via GC–MS demonstrated a high degree of reproducibility, with consistent compound retention times and mass spectra across replicates.

## General notes and troubleshooting

1. Sterility precautions: To prevent contamination, ensure all glassware, media, and reagents are sterilized before use.

2. DNA extraction products can be stored at -20 °C before sequencing.

3. Accurate measurement of reagents is essential for reproducibility.

4. If no microbial growth is observed, verify sterility of ISP2 medium and incubation conditions. Minor fluctuations in temperature (±2 °C) or humidity may affect microbial growth. Consistent incubation conditions are recommended for reproducibility.

5. Genetic variability: Differences in strain genetics may impact secondary metabolite production. If expected results are not obtained, validate the strain identity using 16S rRNA sequencing before proceeding.

6. Reagent preparation: Always prepare fresh sodium hypochlorite and ethanol solutions for surface sterilization to maintain efficiency.

7. ISP2 medium composition: The composition of ISP2 agar may vary by manufacturer, potentially influencing microbial growth. If growth is inconsistent, verify media components and adjust pH to 7.2 ± 0.2 before sterilization.

**Troubleshooting**

Problem 1: Poor or no growth of *Streptomyces* sp. on ISP2 medium.

Possible cause: Improper sterilization of plant material led to contamination.

Solution: Optimize surface sterilization by adjusting ethanol and sodium hypochlorite exposure times (see General Note 4). Ensure all steps are performed under aseptic conditions.

Possible cause: Medium pH is outside the optimal range for *Streptomyces* sp.

Solution: Adjust the pH to 7.2 ± 0.2 before autoclaving to maintain optimal growth conditions.

Problem 2: Low yield of genomic DNA.

Possible cause: Incomplete cell lysis during CTAB extraction.

Solution: Increase the incubation time at 65 °C during lysis and ensure the addition of RNase A to remove RNA contamination.

Problem 3: No secondary metabolite detection in GC–MS analysis.

Possible cause: Poor extraction efficiency.

Solution: Increase the extraction time (24–48 h) and ensure vigorous shaking during solvent extraction (see General Note 3).

Possible cause: Metabolites degraded due to improper storage.

Solution: Store crude extracts at -20 °C in amber vials to prevent degradation by light or oxidation.

Problem 4: Inconsistent antibacterial activity in bioassay.

Possible cause: Variation in crude extract concentration.

Solution: Use a rotary evaporator under controlled vacuum and temperature (54 °C, 80 rpm) to ensure uniform concentration before testing.

Possible cause: Inconsistent agar diffusion well sizes.

Solution: Use a standardized sterile cork borer (6 mm diameter) to create uniform wells for antibiotic diffusion.
